# Processionary Moth Exposure in Europe: Toxic–Irritant and Immunoglobulin E‐Mediated Endotypes, Diagnosis and Management

**DOI:** 10.1111/cea.70415

**Published:** 2026-08-05

**Authors:** Prasad Dasari, Josefine Haas, Wolfgang Rohe, Kai Gloyna, Christoph Viebahn, Susann Forkel, Michael Szardenings, Christine Stadelmann, Michael P. Schön, Timo Buhl

**Affiliations:** ^1^ Department of Dermatology, Venereology and Allergology University Medical Center Göttingen Göttingen Germany; ^2^ Faculty of Natural Resource Management HAWK HHG‐University of Applied Sciences and Art Göttingen Germany; ^3^ Division of Infectious Diseases, Department 3 State Office for Health and Social Affairs Mecklenburg‐Vorpommern Rostock Germany; ^4^ Institute of Anatomy and Cell Biology University Medical Center Göttingen Göttingen Germany; ^5^ Department of Dermatology, Venereology and Allergology University Hospital Leipzig Leipzig Germany; ^6^ Fraunhofer Institute for Cell Therapy and Immunology Leipzig Leipzig Germany; ^7^ Department of Neuropathology University Medical Center Göttingen Göttingen Germany; ^8^ Lower Saxony Institute of Occupational Dermatology University Medical Center Göttingen Göttingen Germany

**Keywords:** allergic sensitisation, caterpillar dermatitis, IgE‐mediated hypersensitivity, processionary moth, setae‐induced inflammation

## Abstract

Processionary moths (*Thaumetopoea* spp.), particularly the oak processionary moth (*Thaumetopoea processionea*), are an increasingly relevant cause of allergic and toxic disease in Europe, occurring in the context of reported range expansion, changing environmental conditions and persistent contamination with airborne urticating hairs (setae). Clinical manifestations range from toxic–irritant dermatitis and ocular injury to respiratory symptoms, while a subset of exposed individuals develops genuine IgE‐mediated disease, including rhinoconjunctivitis, asthma, and rare cases of anaphylaxis. These presentations reflect two mechanistically distinct endotypes: (i) toxin‐driven epithelial penetration by the urticating hairs with neuro‐inflammatory activation and (ii) allergen‐specific sensitisation to defined molecular components such as Tha p 1 and Tha p 2. Accurate endotype differentiation is clinically relevant, as symptom latency, severity, and risk of systemic progression differ substantially. Diagnostic evaluation relies on exposure history and phenotype recognition, complemented in selected cases and experienced centres by skin testing using research‐grade extracts and emerging component‐resolved approaches. Basophil activation testing may provide additional mechanistic insights in selected cases. Management comprises prompt decontamination, anti‐inflammatory therapy, urgent ophthalmologic assessment when eye involvement is suspected, and guideline‐based management of anaphylaxis. The environmental persistence of setae and recurrent seasonal exposure highlight the need for improved surveillance, validated molecular diagnostics, and translational research defining immune mechanisms and biomarkers.

## Highlights

- Most reactions are toxic–irritant, driven by epithelial injury and neuro‐inflammatory activation.
- IgE‐mediated allergy is less common but may cause immediate systemic reactions.
- Endotype differentiation guides diagnosis, risk stratification and management.

## Graphical abstract

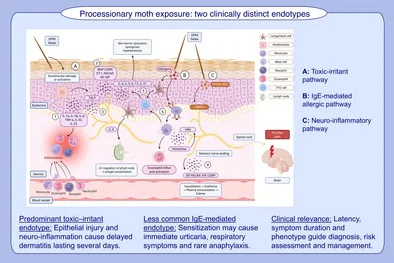

## 1 Introduction

Processionary moths (*Thaumetopoea* spp.)—notably *T. processionea* (oak), *T. pityocampa* (pine), and *T. pinivora* (northern pine processionary moth)—have emerged as a relevant public health concern in Europe in the context of reported range shifts and increasing human exposure [1, 2, 3, 4]. Reported “range expansion” primarily refers to documented changes in geographic distribution rather than to standardised continent‐wide population counts. Long‐term quantitative data on absolute population size or density across Europe are limited and largely derive from regional monitoring programmes with heterogeneous methodologies. Processionary moths are named for their characteristic head‐to‐tail larval processions, which facilitate thermoregulation and collective defences [5, 6, 7]. While previously considered forestry pests, these species now pose direct clinical challenges, particularly as their airborne urticating hairs (“setae”) contain thaumetopoein and other bioactive proteins that can induce a range of toxic and allergic reactions in humans [8, 9, 10, 11, 12, 13]. This review focuses on clinical phenotypes, diagnostic challenges, and management strategies relevant to clinical allergists and dermatologists, with particular emphasis on distinguishing between toxin‐driven irritant reactions and IgE‐mediated hypersensitivity. An endotype‐based perspective is applied to support clinical phenotyping, risk stratification—including anaphylaxis—and interpretation of emerging molecular diagnostic approaches in the context of changing environmental exposure. Most included data derive from case reports, small case series, poison‐control registries, and regional surveillance rather than randomised or prospective controlled studies. Accordingly, we indicate where conclusions are supported by consistent clinical observations and where findings remain hypothesis‐generating and require further validation.

## 2 Methods

This narrative review is based on a non‐systematic literature search of PubMed/MEDLINE and Google Scholar, supplemented by manual screening of reference lists from relevant articles. The search focused on processionary moth exposure in Europe, particularly *T. processionea* and *T. pityocampa*, and covered clinical manifestations, pathophysiology, allergen characterisation, diagnosis, management, and public health aspects. Priority was given to per‐reviewed clinical studies, case series, mechanistic studies, and relevant guideline or surveillance publications. Because the available evidence is heterogeneous and consists largely of case reports, small observational studies, poison‐centre data, and regional surveillance reports, this article should be interpreted as a narrative review rather than a systematic review.

## 3 Results

### 3.1 Ecology, Distribution and Exposure Context of Processionary Moths

The order Lepidoptera comprises butterflies and moths, representing one of the most diverse insect groups with approximately 160,000 species described worldwide [14, 15]. In Lepidoptera, processionary moths of the genus *Thaumetopoea*—notably *T. processionea* (oak), *T. pityocampa* (pine), and *T. pinivora* (northern pine)—are distributed across Europe, Northern Africa, and the Middle East [16, 17, 18]. These species have expanded their range in recent decades, with *T. processionea* (oak processionary moth, OPM) now established throughout southern and northern Europe, including the United Kingdom, from the Black Sea to the Iberian Peninsula following accidental introduction and subsequent spread, likely influenced by multiple ecological drivers [4, 17, 19, 20, 21, 22, 23, 24, 25]. *T. pinivora* has similarly extended northward around the Baltic Sea, Germany, and Poland [26, 27]. Detailed European distribution patterns have previously been illustrated by Godefroid et al. [28].

Over the last decades, *T. processionea* has expanded markedly across Central and Western Europe, facilitated by milder winters, warmer springs, international plant trade, and urban heat islands [2, 16, 17, 22, 29, 30, 31, 32]. Once uncommon before 1900 [22, 33, 34, 35, 36, 37], it is now established in Belgium, the Netherlands, Germany, and since 2006 also in the United Kingdom following accidental introduction via imported plants [28, 30]. The species increasingly colonises urban and suburban oak trees, where warmer microclimates support larval survival and promote human exposure [30, 38]. While northward and altitudinal range shifts have been associated with milder winters and warming trends in several modelling and observational studies [16, 17, 30], alternative or complementary explanations have been proposed. These include historical underreporting of populations, land‐use changes, urban heat islands, and long‐distance dispersal via plant trade or human transport [22, 26, 34]. Genetic analyses and historical records suggest that some newly documented populations may represent re‐emergence or previously unrecognised persistence rather than exclusively recent climate‐driven expansion.

Among the native European oak species, *T. processionea* caterpillars preferentially infest pedunculate oak ( *Quercus robur* ) and, to a lesser extent, sessile oak ( *Q. petraea* ), which are the dominant oak species in Central and Western Europe [12, 39]. Within *Q. robur* , individual trees and populations exhibit variable susceptibility, with some trees showing lower rates of caterpillar colonisation and damage [39, 40]. This resistance is linked to constitutive chemical defences, particularly higher concentrations of polyphenolic compounds such as flavonoids (kaempferol, quercetin glucosides) and condensed tannins, which are associated with reduced herbivore performance and feeding [39, 40]. While most literature focuses on native European oaks, the genus Quercus as a whole is susceptible to infestation, as the moth does not exhibit strict host specificity within the genus [12].

Larvae of *T. processionea* feed gregariously on oaks and build communal silk nests that contain high concentrations of urticating setae (details below) [1, 5, 17, 22]. These setae readily become airborne, adhere to surfaces, and can cause toxic–irritant dermatitis, conjunctivitis, and non‐IgE–mediated inflammatory reactions in humans, as well as veterinary health problems in companion animals and livestock [1, 5, 23]. The setae are easily released from the larvae and their nests, rather than remaining strongly adhered to the body surface or within the silk structure. They are adapted for easy detachment and dispersal by passive mechanical mechanisms, specifically the presence of predetermined breaking points at the base of the setae [1, 41, 42]. The nests themselves, constructed from silk threads, do not exhibit strong adhesion to the setae; instead, the setae are readily dislodged by mechanical disturbance or air currents, thus facilitating environmental contamination and human contact [1, 41, 42].

### 3.2 Clinically Relevant Life Cycle

**FIGURE 1:**
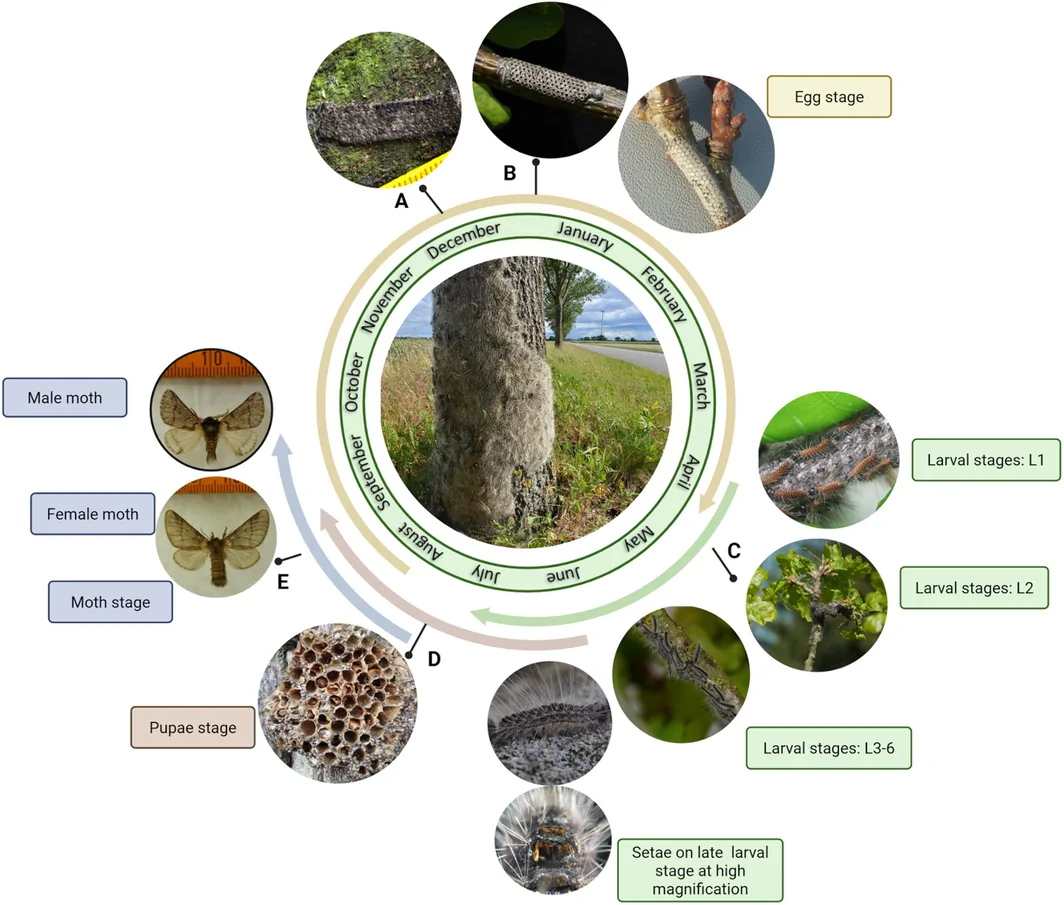
Life cycle of the oak processionary moth (*Thaumetopoea processionea*, OPM). (A) Egg mass covered with abdominal scales deposited by the female moth. (B) Two examples of intact overwintering egg masses on oak twigs. Embryonic development occurs before winter, and first‐instar larvae remain within the eggs until hatching in spring. (C) Larval development from April to June, showing early instars L1 and L2 and late instars L3–L6. The close‐up image shows the dorsal fields of urticating setae in a late‐instar larva; these setae develop from the third instar onwards. (D) Empty pupal cases remaining within nest material after adult emergence. (E) Adult male and female moths, which emerge mainly from August to September, mate and deposit egg masses, completing the univoltine life cycle. The seasonal timing shown reflects typical conditions in Central Europe. Macro‐images were obtained by the authors; schematic elements were created using BioRender.

The life cycle of the oak processionary moth (*T. processionea*) is closely linked to seasonal patterns of human exposure and clinical risk (Figure 1).

Egg Stage: Adult females lay egg masses (50–300 eggs) in oak canopies between August and September and cover them with abdominal scales (Figure 1A) [6, 17]. Embryos develop into first instar (L1) larvae by autumn's end [12, 17]. The overwintering stage shows marked cold tolerance, with survival at very low winter temperatures reported (Figure 1B) [43]. Eggs hatch in spring (April–May), synchronised with oak leaf emergence [17]; if leaf flushing is delayed, larvae may enter embryonic diapause and survive several weeks of starvation [17]. Egg masses themselves are not clinically relevant but mark areas of potential subsequent larval infestation and exposure.

Larval Stage: Larvae progress through six instars (L1–L6) until pupation [5]. Early instars feed on young foliage and construct silk nests. Early instars carry no or very few urticating setae and are therefore of lower clinical relevance. From the third instar onwards, larvae develop dense urticating setae, representing the phase of highest dermatologic and allergic risk. Now, the larvae move in characteristic head‐to‐tail “processions” primarily for collective defence and orientation (Figure 1C) [5, 6, 7]. Critically, specialised urticating setae develop from the third to sixth instars, concentrated on dorsal abdominal segments (“mirrors” or “dorsal tubercles”) [1, 6, 7, 41]. These setae are continuously released and responsible for the majority of human health effects. Communal nests serve as reservoirs of shed setae, which may remain airborne and clinically active even in the absence of visible larvae.

Pupal stage: Fully grown L6 larvae pupate within nests from late June, with pupation lasting two to three weeks [5]. Empty pupal cases remain within the nest material after adult emergence (Figure 1D). Some pupae remain in diapause, delaying emergence for one or more years [5, 7, 41, 44, 45]. Although pupae themselves are not directly toxic, residual setae within cocoons may remain a source of exposure. Setae from moulted skins, abandoned nests, and contaminated surfaces persist in the environment, dispersing by wind or human activity and remaining biologically active for up to ten years [11, 12, 41, 45].

Adult stage: Adult moths emerge from August to September, influenced by temperature and precipitation (Figure 1E) [7]. They are nocturnal, live for three to four days, and do not possess setae. After emergence, adults mate, and females lay egg clusters, restarting the univoltine cycle. Adults do not feed, with the entire stage completed in about one week [5, 6, 7, 17, 43, 44]. Adult moths do not possess urticating setae and are generally not considered clinically relevant.

### 3.3 Setae: Structural Basis of Allergenic and Toxic Effects

**FIGURE 2:**
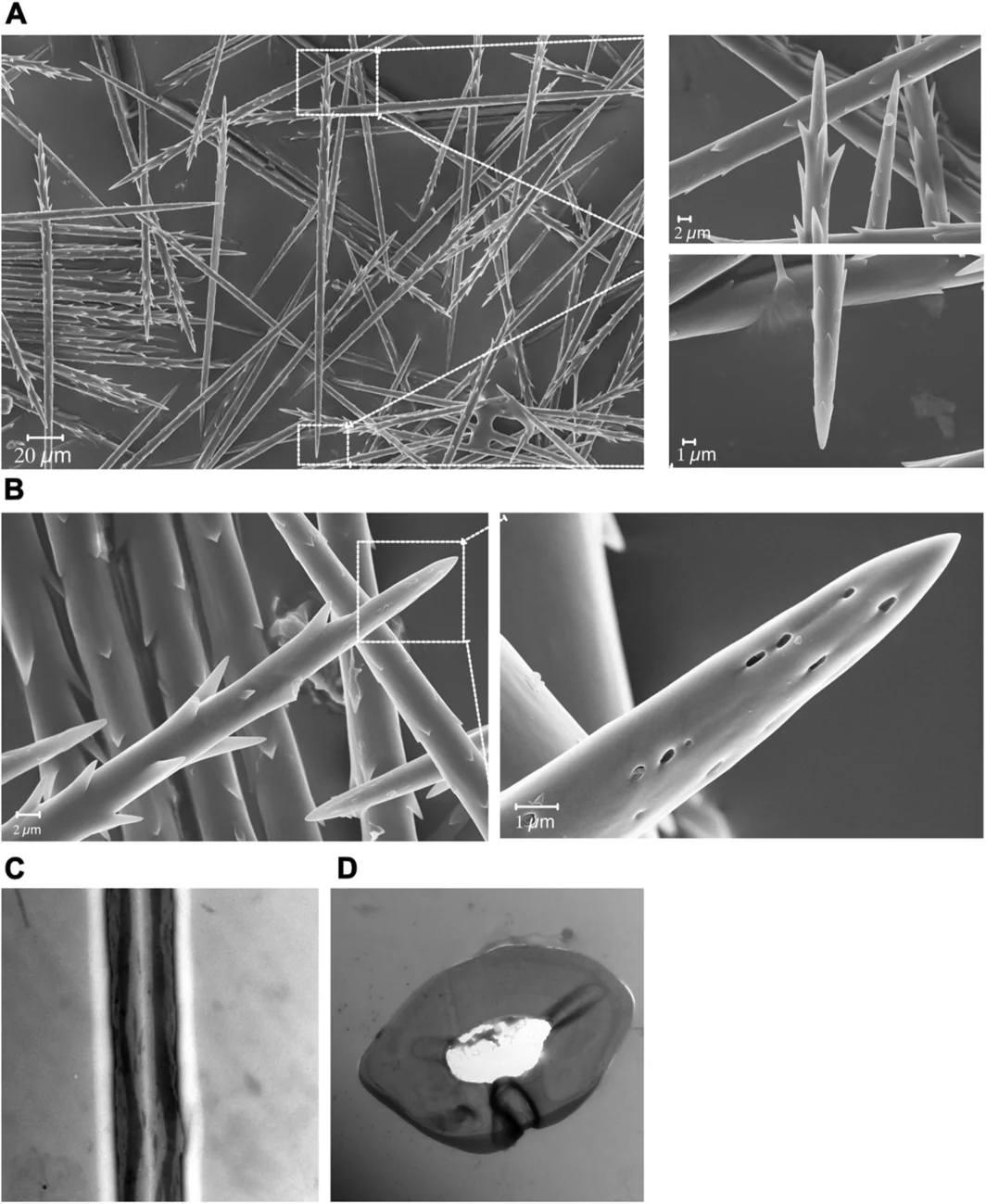
Ultrastructural features of *T. processionea* urticating setae. Conventional scanning electron microscopy reveals the characteristic chitinous structure of setae with distally oriented barbs predominantly located on the distal parts of the shafts (A, B); numerous small openings are found at the distal ends (B; right panel) and a hollow internal core (C, cross‐section; D, longitudinal section). For panel A + B, images were obtained by conventional scanning electron microscopy prepared from late‐instar larvae fixed on a grid (Institute of Anatomy and Cell Biology). For panel C + D, setae were embedded in Renlam M1 and ultrathin sections (70–80 nm) were prepared using a Leica UC7 ultramicrotome. The images were obtained using an EM 10C transmission electron microscope, Zeiss (Department of Neuropathology).

The urticating setae of processionary moth larvae are hollow, chitinous structures (150–250 μm long, 5–10 μm in diameter, with 1–2 μm thick walls) that provide remarkable environmental persistence and are central to their clinical effects [1, 7, 9, 12, 41]. Setae are actively released when larvae are disturbed [1, 41]. Scanning electron microscopy reveals distally oriented barbs on the shaft and numerous small pores at the distal end, while longitudinal and cross‐sectional analyses confirm hollow interiors with a diameter of 1–2 μm (Figure 2). Setae facilitate penetration into skin, mucous membranes, and ocular tissues while the barbs prevent easy removal, thereby prolonging tissue contact and enhancing delivery of urticating compounds [7, 45]. The hollow core stores toxins and allergens, predominantly thaumetopoein [9]. However, the functional relevance of the distal pores remains incompletely understood. Thaumetopoein and related bioactive proteins have been described predominantly in *T. pityocampa*; however, species‐specific toxin profiles for *T. processionea* remain incompletely characterised.

**FIGURE 3:**
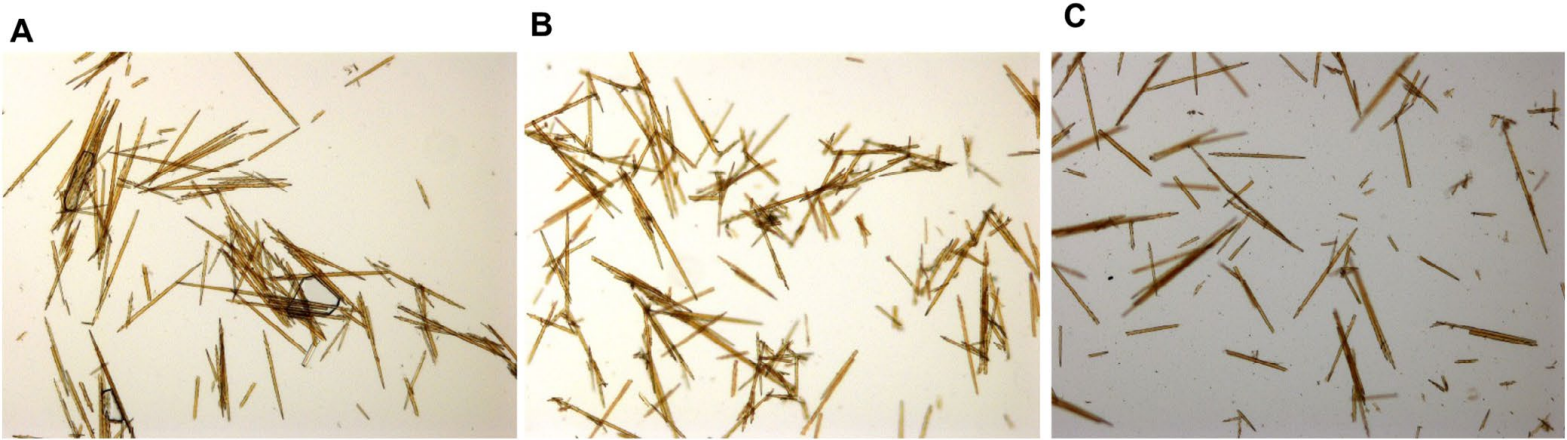
Mechanical resilience of *T. processionea* urticating setae. Representative micrographs obtained after experimental mechanical stress testing. (A) Repetitive sonication at 70 cycles for 30‐s intervals using Bandelin Sonopuls HD70 (six repetitions), with most setae remaining intact. (B) Continued sonication of the same setae at 80 cycles for 60‐s intervals (six repetitions), showing preserved structural integrity. (C) Subsequent homogenisation using metal beads (5 mm diameter) with a tissue homogeniser (TissueLyser LT, Qiagen) at 50 oscillations per second. The images were acquired using an Axioskop 2, Zeiss. Across all sequential treatments, setae largely retained their structure, indicating pronounced resistance to mechanical damage.

Experimental analyses demonstrate a pronounced mechanical resilience of setae (Figure 3). Despite sonication and metal‐bead agitation, most remained structurally intact. Their durability likely contributes to long‐term biological activity and environmental persistence; setae have been reported to remain biologically active for years, in some reports for up to a decade, posing a sustained public‐health concern due to prolonged exposure risks.

Upon epithelial contact, setae penetrate unidirectionally and anchor into tissues, causing mechanical irritation and foreign‐body reactions. Embedded fragments are typically surrounded by mixed inflammatory infiltrates rich in neutrophils, eosinophils, and lymphocytes [44]. Beyond mechanical trauma, biochemical compounds released from the lumen may activate inflammatory and neurogenic pathways, forming the mechanistic basis for subsequent toxic–irritant and allergic reactions [9, 13].

### 3.4 Allergy and Other Health Implications

Exposure to processionary moth setae can lead to a broad spectrum of cutaneous, respiratory, ocular, and systemic symptoms. The underlying mechanisms include both direct toxin‐induced inflammation and IgE‐mediated allergy (Table 1). Clinically, cutaneous reactions predominate [44, 51, 52, 66, 67], while respiratory and ocular involvement occur less frequently but may be severe [45, 57, 58, 65, 67, 68]. These two mechanistic pathways differ in latency, immunologic features, and clinical course, and may coexist within the same individual. Table 1 compares the underlying mechanisms, clinical features, diagnostic approaches, treatment, and prognosis of toxic–irritant and IgE‐mediated endotypes following *Thaumetopoea* exposure.

**TABLE 1:** Clinical phenotypes in relation to immunological endotypes of Oak Processionary Moth exposure.

Category	Toxic‐Irritant Endotype	IgE‐mediated endotype (Type I)	References
Pathophysiology	Mechanical penetration of setae; release of thaumetopoein and pro‐inflammatory proteins; neurogenic inflammation (experimental evidence suggests involvement of TRPV1); non‐IgE mast‐cell/basophil activation	Sensitisation to defined allergens (e.g., Tha p 1, Tha p 2); IgE‐mediated immediate hypersensitivity with mast‐cell/basophil degranulation	[9, 10, 13, 46, 47, 48]
Latency	Typically hours, occasionally up to 12–24 h after exposure	Minutes to ≤ 1 h after exposure	[44, 49]
Skin manifestations	Papulovesicular dermatitis, urticarial papules, eczematous‐like lesions	Urticaria, angioedema, rapidly developing wheals after contact	[44, 50, 51]
Duration of cutaneous manifestations	Usually several days	Usually hours to < 24 h, although overlap may occur	[44, 49, 50]
Respiratory involvement	Irritation of upper airways, cough; rarely bronchial symptoms	Allergic rhinitis, bronchospasm or asthma exacerbations in sensitised individuals	[11, 12, 52]
Ocular involvement	Mechanically induced keratoconjunctivitis; foreign‐body reaction; ophthalmia nodosa due to intraocular setae migration	Allergic conjunctivitis (may coexist with toxic effects)	[53, 54, 55, 56, 57, 58]
Systemic reactions	Rare; mostly nonspecific toxic symptoms	Generalised urticaria, rare cases of anaphylaxis, mainly reported after intense or repeated exposure	[59, 60]
Diagnostics	Exposure history; detection of setae; no specific IgE	SPT with non‐standardised in‐house extracts (no validated allergen quantification or non‐irritant concentrations; research use only); specific IgE testing to processionary moth allergens (not standardised and not commercially available); BAT, if available	[12, 47, 49, 61, 62]
Therapeutic approach	Decontamination; topical/systemic corticosteroids; antihistamines; ophthalmologic care when needed	Guideline‐based allergy management; antihistamines; inhaled therapy for asthma when indicated; epinephrine for anaphylaxis	[58, 63]
Typical patient groups	General population outdoors; children; incidental exposure	Repeated exposure: Forestry workers, municipal staff, outdoor professionals	[11, 64]
Prognosis	Usually self‐limiting; ocular complications possible with deep setae migration	Recurrent symptoms with re‐exposure; risk of severe reactions in sensitised individuals	[44, 65]

#### 3.4.1 Pathophysiology and Clinical Manifestations of Setae‐Induced Reactions

The pathophysiology of processionary moth exposure is mediated by both chemical and mechanical mechanisms [13, 46, 49, 50, 69]. Current knowledge on clinical manifestations and organ involvement is largely based on retrospective case series, poison‐control centre data, and small observational cohorts, often without standardised exposure assessment or control groups. Reported frequencies of cutaneous, respiratory, ocular, and systemic disease should therefore be interpreted as approximate and context‐dependent rather than as precise incidence estimates. Available quantitative data derive primarily from small observational cohorts. In a paediatric survey conducted in a pine woodland region of Spain, 9.2% of interviewed children reported cutaneous symptoms after exposure to pine processionary caterpillars, and an IgE‐mediated mechanism confirmed by skin testing and immunoblotting was documented in 4 of 59 evaluable symptomatic cases (6.8%) [70]. These figures reflect a selected regional paediatric population and should not be interpreted as population‐based incidence estimates. In poison‐centre registries, most reported events are classified as toxic–irritant manifestations, while systemic immediate reactions are uncommon [67]. The hollow setae release proteinaceous toxins such as thaumetopoein upon rupture [9]. Proteotranscriptomic analyses of *T. processionea* setae have identified over 200 proteins, including serine proteases, lipocalins, and chitinases with immunomodulatory potential [10]. In a recent experimental ex vivo study, setae extracts have been shown to induce dose‐dependent basophil activation and to modulate transient receptor potential vanilloid 1 (TRPV1) signalling pathways on sensory neurons; however, the clinical relevance of this finding remains to be established [13].

**FIGURE 4:**
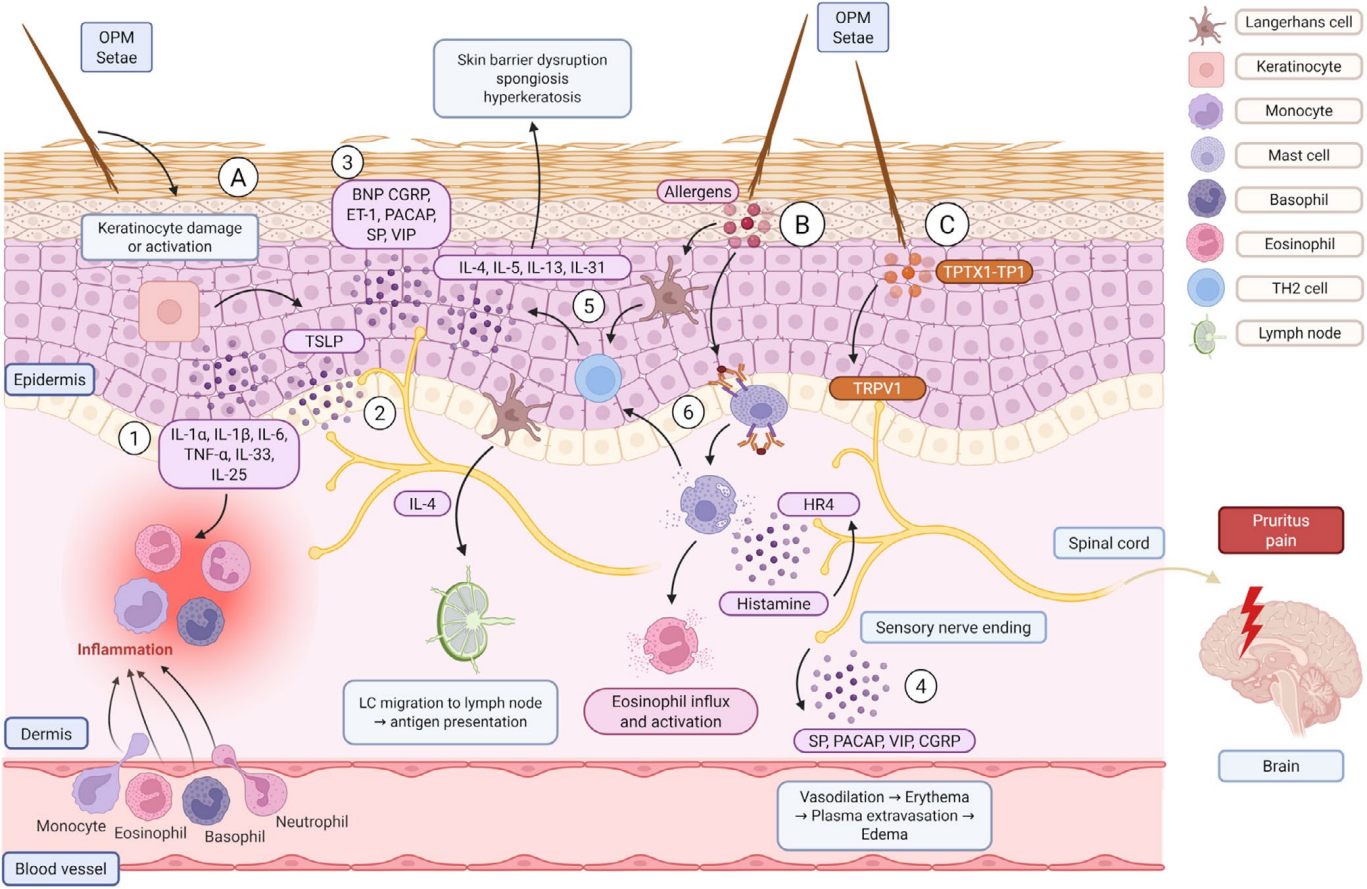
Hypothesis‐driven model of the immunological cascade following setae‐induced skin contact and allergic sensitisation. (A) Setae penetration disrupts the epidermal barrier and damages keratinocytes, inducing alarmin release (1) and recruitment of innate immune cells. Keratinocyte‐derived TSLP activates sensory neurons via the TSLP receptor/TRPA1 axis, inducing itch (2), and promotes Th2‐skewed inflammation with recruitment of eosinophils, mast cells, and basophils. Activated sensory neurons release neuromediators (e.g., SP, CGRP, PACAP, VIP, endothelin‐1) that amplify inflammation and vasodilation, enhance plasma extravasation, and promote further leukocyte recruitment (3, 4). (B) Repeated setae exposure can induce adaptive immune responses with Th2‐driven allergic sensitisation (5), characterised by mast cell degranulation, histamine release, eosinophil activation, and increased Th2 cytokine production (6). (C) Persistent pruritus is mediated by histamine, Th2 cytokines, and other pruritogens via histamine‐dependent and ‐independent pathways, contributing to chronic allergic dermatitis. The setae toxin TPTX1 has been shown to modulate TRPV1 signalling in processionary species. Abbreviations: SP, substance P; CGRP, calcitonin gene‐related peptide; PACAP, pituitary adenylate cyclase‐activating polypeptide; VIP, vasoactive intestinal peptide. This figure is an original schematic illustration based on mechanisms discussed in Steinhoff et al. [71]. Created with BioRender.

Initial exposures commonly provoke toxic–irritant inflammation through non‐specific mast‐cell and basophil activation, leading to histamine release independent of IgE. IgE‐mediated (Type I) hypersensitivity has been documented in sensitised individuals following exposure to processionary caterpillars, with reports of immediate‐type reactions and demonstrable specific IgE to defined caterpillar allergens [46, 47, 49, 61]. The proposed sequence of epithelial injury, neuro‐immune activation, and Th2‐driven allergic sensitisation is summarised in Figure 4. Together, these mechanisms underlie the wide clinical spectrum of setae‐induced disease (Table 2).

**TABLE 2:** Clinical manifestations associated with oak processionary moth exposure, with corresponding exposure routes and underlying mechanisms.

Clinical manifestation	Typical exposure routes	Suspected underlying mechanisms	References
Papulovesicular dermatitis/urticarial papules	Direct skin contact; contaminated clothing; airborne setae settling on skin	Mechanical penetration of barbed setae; thaumetopoein‐induced toxic inflammation; neurogenic inflammation including TRPV1 activation; non‐IgE mast‐cell activation; IgE‐mediated wheal formation in sensitised individuals	[44, 50, 52, 66]
Intense pruritus/eczematous plaques	Direct skin contact; indirect contact with nests or contaminated surfaces	Neurogenic inflammation via TRPV1; toxins/proteases; foreign‐body reaction; eczematous‐like inflammatory response	[10, 13, 44, 50]
Angioedema/generalised urticaria	Direct or airborne exposure; high‐dose exposure	IgE‐mediated immediate reaction in sensitised patients; widespread mast‐cell activation	[44, 49]
Rhinorrhea, nasal irritation, cough	Airborne exposure (inhalation of setae)	Mechanical mucosal irritation; toxic–irritant inflammation; possible contribution of neurogenic inflammation (TRPV1)	[11, 12, 50, 69]
Asthma‐like symptoms/bronchospasm	Inhalation of airborne setae; occupational exposure	Toxic airway irritation; IgE‐mediated bronchial hyperreactivity in sensitised individuals	[11, 12, 52]
Conjunctivitis/keratitis/foreign‐body sensation	Airborne or direct ocular exposure	Mechanical penetration of setae; toxic–inflammatory response; granulomatous reaction	[42, 44, 57, 58, 65]
Intraocular migration, uveitis, vitritis, secondary glaucoma, focal cataract	Direct or airborne ocular exposure	Unidirectional migration of barbed setae; persistent foreign‐body inflammation; intraocular granuloma formation (ophthalmia nodosa)	[53, 54, 55, 57, 58]
Oropharyngeal edema/gastrointestinal discomfort	Accidental ingestion (children)	Toxic mucosal irritation; local mast‐cell activation; rarely IgE‐mediated systemic response	[44, 60]
Anaphylaxis	High‐dose inhalation or direct contact; occupational exposure	IgE‐mediated systemic mast‐cell degranulation reported in rare case reports, including high‐exposure settings	[59, 60]

*Note:* The table summarises major organ‐specific clinical presentations caused by urticating setae of *Thaumetopoea processionea*, linking each manifestation to typical exposure routes (cutaneous, airborne, ocular, ingestion, occupational high‐dose exposure) and the predominant underlying mechanisms. Mechanisms include mechanical penetration of barbed setae, toxin‐induced and neurogenic inflammation including TRPV1 activation, non‐IgE mast‐cell/basophil activation, and IgE‐mediated immediate hypersensitivity in sensitised individuals.

##### 3.4.1.1 Clinical Spectrum

**FIGURE 5:**
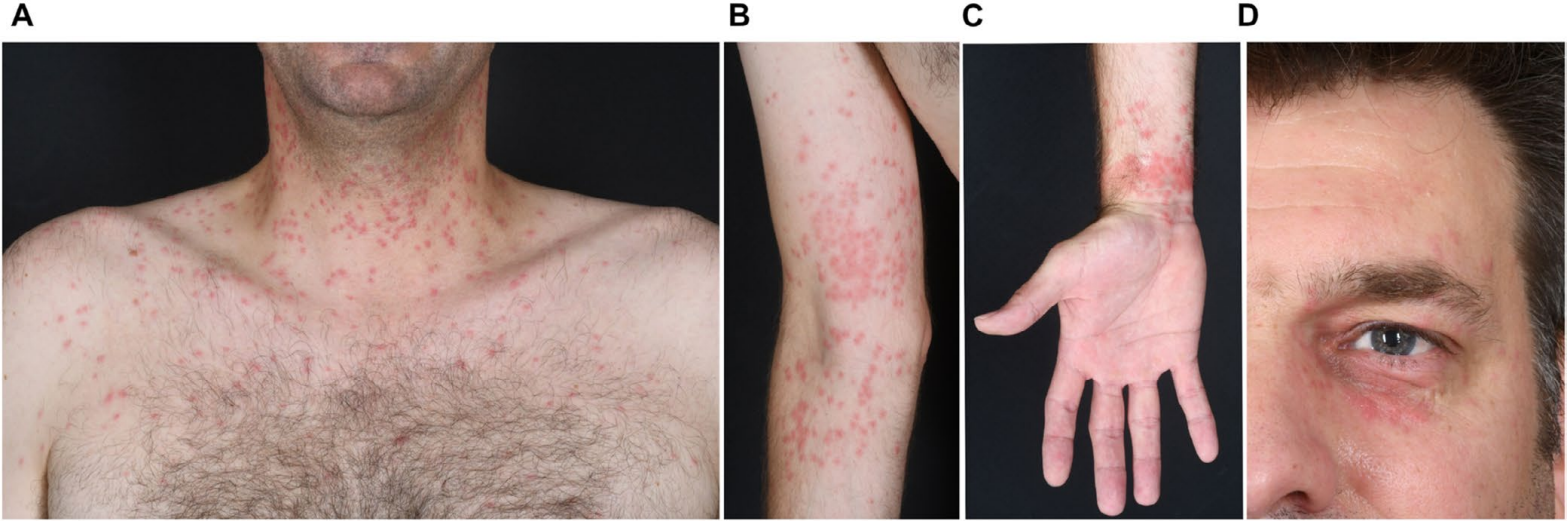
Cutaneous and ocular manifestations following exposure to *T. processionea* setae. Clinical images show caterpillar dermatitis with erythematous papules and vesicles on exposed skin areas, including the neck (A), forearm (B), and wrist (C), consistent with direct or airborne setae contact. (D) Conjunctivitis with periorbital urticaria and erythema after setae exposure. All images were obtained approximately 20 h post‐exposure. Clinical photographs were obtained during routine clinical assessment at the Department of Dermatology. The patient provided written informed consent for publication of clinical images.

Cutaneous: Direct skin contact causes erythema, urticarial wheals, and papulovesicular eruptions (“caterpillar dermatitis”), often with severe pruritus (Figure 5) [44, 50, 51, 52, 66, 67]. Symptoms usually begin 1–12 h after exposure; immediate‐onset reactions within minutes have been reported in sensitised individuals, mainly in *T. pityocampa* allergy cohorts [49, 61]. Toxic–irritant papular or eczematous lesions typically persist for several days, whereas IgE‐mediated wheals and urticaria usually resolve within hours or approximately one day. In sensitised patients, a duration of less than 24 h has been associated with IgE‐mediated reactions, although overlap between the endotypes limits the diagnostic specificity of symptom duration [44, 49, 50].

Respiratory: Inhalation of airborne setae may cause cough, sore throat, and dyspnoea [11, 12, 50, 69]. Severe cases can present with bronchial hyperreactivity or asthma‐like symptoms, especially in occupational settings [11, 12, 52].

Ocular: Ocular involvement is the most serious due to the risk of permanent visual impairment [45, 57, 58, 65, 67]. Setae can penetrate the conjunctiva or cornea, causing foreign‐body sensation, photophobia, tearing, and redness. Retrograde‐barbed spines of the setae may drive progressive intraocular migration [53, 54, 55, 56, 57, 58]. Persistent or deep penetration may lead to keratitis, anterior uveitis, secondary glaucoma, or cataract formation [45, 57, 58, 65, 72].

Systemic: Systemic reactions are uncommon and documented mainly in isolated case reports. Reported manifestations include generalised urticaria, angioedema, bronchospasm, and rare cases of anaphylaxis after intense, repeated, or occasionally oral exposure [59, 60]. Both IgE‐dependent and non‐IgE mechanisms may contribute.

Differential diagnoses for the cutaneous, respiratory, and ocular presentations include arthropod assaults, *Toxicodendron* dermatitis, papular urticaria, irritant conjunctivitis, and, in ocular cases, herpes zoster ophthalmicus. Unlike these conditions, processionary‐moth exposure typically presents with clusters of intensely pruritic papules after outdoor activities near infested trees, often accompanied by a foreign‐body sensation due to retained setae. Symptom latency—minutes in IgE‐mediated reactions vs. hours in toxic–irritant disease—provides an additional distinguishing feature. Identification of setae fragments on the skin or ocular surface, when present, supports the diagnosis and helps discriminate processionary‐moth–associated disease from other causes of dermatitis or conjunctivitis.

#### 3.4.2 Clinical Phenotypes and Immunological Endotypes of Processionary Moth Exposure

An immediate‐type (Type I) endotype is supported by sensitisation data and molecular allergology. In selected cohorts with suggestive histories, skin‐prick testing using non‐standardised in‐house extracts to *T. pityocampa* has shown positive results [49]; however, standardised extracts and validated concentrations are currently lacking, and irritant reactions cannot be fully excluded. Latency to symptoms is shorter in sensitised individuals. Systemic immediate reactions, including anaphylaxis, have been reported only rarely and are documented primarily in isolated case reports. Component characterisation identified Tha p 1 as a major allergen [46], and subsequent work delineated Tha p 2 as another major component recognised by patient IgE [47, 48]. Recombinant Tha p 2 has been evaluated in research‐based immunoassays demonstrating specific IgE binding; however, this does not represent a validated, standardised component‐resolved diagnostic assay [47]. Molecular allergens such as Tha p 1 and Tha p 2 have been well characterised for *T. pityocampa*. For *T. processionea*, direct molecular data remain limited, and current assumptions are largely based on homology and indirect evidence suggesting potential cross‐reactivity rather than species‐specific allergen characterisation.

A second, primarily toxic–irritant endotype reflects direct mast‐cell activation and epithelial injury by thaumetopoein and other bioactive components released from setae [9]. This leads to intensely pruritic papular dermatitis (“caterpillar dermatitis”) with grouped urticarial papules or eczematous plaques, occasionally bullous, often after outdoor activities near infested trees or after nest removal [44]. Poison‐centre surveillance corroborates the predominance of cutaneous disease and frequent ocular involvement [67]. Respiratory symptoms occur in a minority and are modulated by wind and mechanical disturbance that amplify airborne exposure [11, 12]. Both endotypes may occur in different individuals, but can also coexist within the same patient. In such cases, toxic–irritant tissue injury may act as an adjuvant‐like trigger that amplifies IgE‐mediated responses in sensitised individuals. A comparable interaction between direct tissue damage and allergen‐specific IgE has been described in other arthropod‐related reactions; however, for processionary moth exposure, this remains a plausible but as yet insufficiently studied hypothesis.

Risk is heterogeneous [73]. Forestry workers, arborists, and municipal staff share high exposure burdens [64]. Occupational series document dermatitis, rhinoconjunctivitis, and asthma, sometimes with work‐exacerbation patterns [49]. Children and bystanders can be affected in suburban outbreaks [11, 12], and rare systemic reactions can occur after ingestion, e.g., through hand‐to‐mouth behaviour in infants [59]. These phenotypes illustrate dose and route effects and reinforce that distinct endotypes—IgE‐dominant vs. toxic–irritant—can coexist within outbreaks, as summarised in Table 1 [64].

Ophthalmia nodosa exemplifies hair‐mediated inflammation across compartments in the eye [53]. Setae may lodge in conjunctiva and cornea but can also migrate intraocularly, provoking anterior uveitis, vitreous inflammation, retinal vasculitis, or foreign‐body reactions [54]. Series and case reports show that symptoms can be deceptively mild initially. Slit‐lamp, optical coherence tomography, ultrasound, or fundus imaging often reveals occult setae [55]. Management hinges on meticulous hair removal when visible [56], topical and/or systemic corticosteroids for inflammation control, and selective vitrectomy for persistent intraocular disease [54].

Evidence appraisal of Tha p 1 and Tha p 2. Early extract‐based studies demonstrated specific IgE binding to multiple caterpillar proteins in sensitised individuals, with several reactive bands detected by immunoblot; however, individual molecular allergens were not identified at that stage [49, 61]. The subsequent isolation and characterisation of Tha p 1 established it as a defined major allergen with confirmed IgE recognition in sensitised patients [46], followed by the description of Tha p 2 as an additional IgE‐binding component evaluated in research‐based diagnostic assays [47, 48]; in the original report, IgE reactivity was demonstrated, whereas IgG binding was not detected by Western blotting [48]. Sequence data for Tha p 2 and homologous sequences in *T. processionea* have also been reported [74].

Thus, Tha p 1 and Tha p 2 represent the best‐characterised molecular allergens of *T. pityocampa* to date. Nevertheless, available data derive from relatively small, selected cohorts, and standardised extracts or commercially available in vitro diagnostic assays are currently lacking. Quantitative relationships between specific IgE levels to *Thaumetopoea* allergens and clinical severity have not yet been systematically defined.

For *T. processionea*, molecular allergen characterisation remains limited. Proteo‐transcriptomic analyses have identified numerous setal proteins [10]; however, these studies did not assess IgE reactivity or clinical allergenicity, and the allergenic relevance of most identified proteins has not yet been established.

### 3.5 Diagnosis and Management From an Allergological Perspective

History should address seasonality (late spring–summer for *T. processionea*; fall/winter for *T. pityocampa* in some regions) [12], geography (near infested oaks/pines) [75], activity (nest removal, groundskeeping), and symptom latency (symptoms within minutes to 1 h suggest a Type I allergic reaction; delayed pruritic papules favour an irritant/toxic reaction) [44]. Ask explicitly about ocular foreign‐body sensation and lower‐airway symptoms [67]. Exposure confirmation is supported by local surveillance or arborist reports during known infestations or outbreaks [12]. Allergy testing strategy should be anchored to clinical likelihood. Where available, skin‐prick testing has been performed using non‐standardised in‐house extracts derived from whole larvae or isolated setae [49]. These preparations lack validated allergen quantification and defined non‐irritant test concentrations. Positive tests have been reported in selected cohorts and were associated with shorter symptom latency and more systemic reactions in occupational settings [49]. However, given the intrinsic toxic–irritant properties of setae, irritant or non–IgE‐mediated reactions cannot be excluded. Skin testing should therefore be confined to experienced centres and is not currently recommended as a routine diagnostic procedure.

Serum‐specific IgE to native extracts is not standardised, but component‐resolved diagnostics are emerging [47]. IgE to Tha p 1 and Tha p 2 supports genuine sensitisation and may help to distinguish allergic endotypes within mixed outbreaks [46, 47, 48]. Recombinant component assays (e.g., Tha p 2) remain research tools and still require rigorous validation [47, 62]. Basophil activation testing may be a helpful adjunct when extract/component quality is assured, and conventional tests are inconclusive [62]. Limitations include lack of commercial in vitro diagnostic (IVD)‐grade reagents and inter‐laboratory variability; thus, interpretation should be integrated with exposure history and phenotype [76]. In suspected anaphylaxis, paired serum tryptase (acute and baseline) follows guideline practice to support mast‐cell activation [63]. Otherwise, there is no routine role for specific provocation testing due to risk and poor standardisation for processionary allergens [77]. The diagnosis of OPM‐associated allergy remains primarily clinical, supported by history and exposure assessment, while advanced immunological assays are mainly restricted to research settings. In the absence of processionary‐moth–specific treatment trials, management follows established guideline‐based approaches. Laboratory investigations and provocation tests should be approached with caution [63].

Acute management follows organ involvement and severity:

- Dermatologic/toxic–irritant disease: Immediate decontamination (clothing removal, showering with soap, avoiding rubbing) [44]. Topical medium‐ to high‐potency corticosteroids and oral H1‐antihistamines relieve pruritus and dermographism [57]. Short systemic corticosteroid tapers are reserved for severe, widespread reactions.
- Allergic rhinoconjunctivitis/asthma: Standard pharmacotherapy with intranasal steroids and/or antihistamines; short‐acting beta‐agonists and inhaled corticosteroids per control, plus exposure avoidance [63].
- Ophthalmia nodosa: Prompt ophthalmologic evaluation for slit‐lamp exploration and hair removal, as retained setae may be easily overlooked and can migrate despite initially mild symptoms; topical steroids and pupil‐dilating agents for kerato‐conjunctival inflammation [45]; escalation to systemic steroids or intraocular surgery for retained setae or persistent posterior‐segment inflammation [53, 54].
  Ocular red flags and indications for urgent referral: Several clinical features warrant same‐day ophthalmologic assessment due to the risk of deep tissue penetration and permanent visual impairment. Red flags include pronounced foreign‐body sensation, photophobia, rapidly increasing ocular pain, visible setae on the conjunctiva or cornea, reduced visual acuity, or new floaters. Persistent or worsening symptoms after initial decontamination should raise concern for deeper migration. Slit‐lamp examination is essential for detecting superficial and mid‐stromal setae; anterior‐segment OCT may help identify deep corneal involvement, whereas ultrasound B‐scan and fundus imaging are appropriate to assess secondary inflammatory changes and posterior‐segment involvement when intraocular migration is suspected, or direct visualisation is limited. Superficial setae should be removed promptly to minimise deeper penetration. Persistent intraocular hairs may require systemic corticosteroids and, selectively, pars plana vitrectomy [53, 54]. Evidence is largely based on small case series, and recommendations therefore reflect expert consensus.
- Anaphylaxis: Adhere to international guidance—prompt intramuscular epinephrine, airway support, oxygen, fluids, adjunct H1/H2 blockers and corticosteroids, observation, and discharge with an auto‐injector and action plan [63].

Prevention and follow‐up extend beyond individual episodes [67]. Counselling should cover seasonality, visual identification of nests, and the importance of personal protective equipment (protective garments, eye protection, and respiratory masks) for exposed workers [44, 64]. Municipalities should implement integrated pest management and safe nest‐removal protocols [78]. Sensitised individuals with systemic reactions warrant an emergency plan and an epinephrine auto‐injector [63]. At present, allergen immunotherapy is not available due to the absence of standardised extracts and limited clinical evidence. Follow‐up should review symptom control, reinforce avoidance, and reassess risk in recurrent seasonal exposure [12].

### 3.6 Public Health and Control Strategies

**FIGURE 6:**
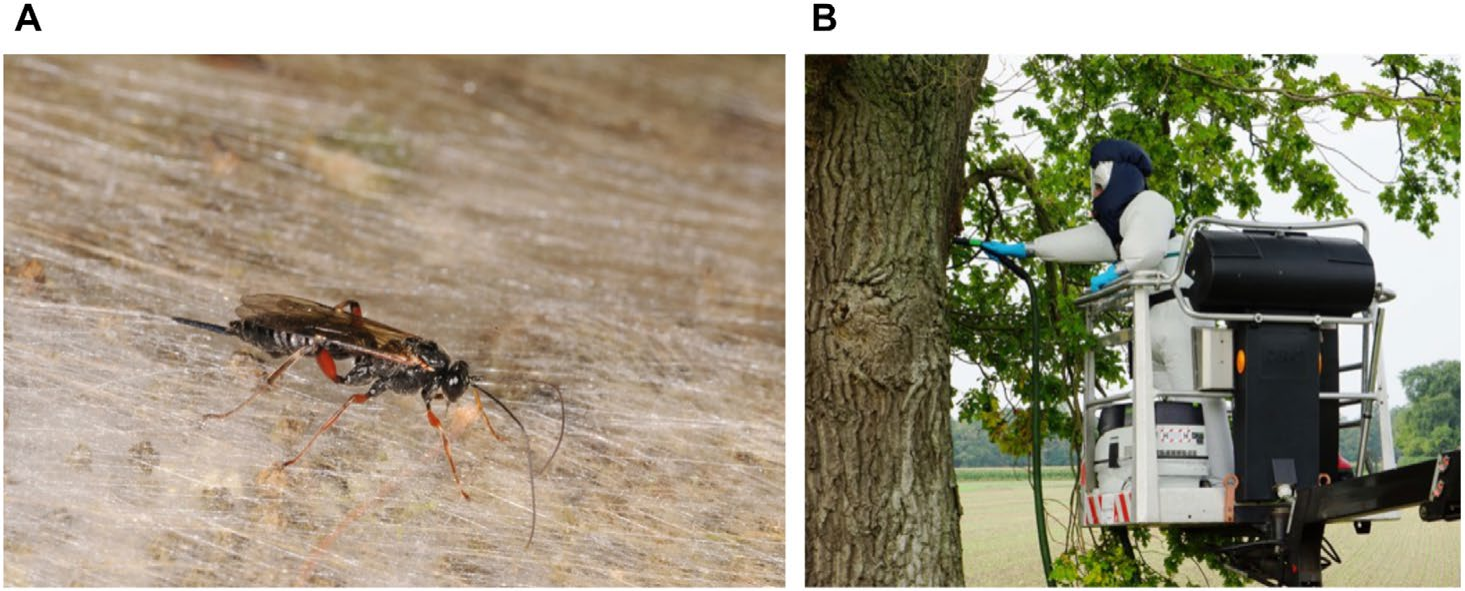
Strategies for oak processionary moth control. (A) A female parasitic wasp (*Ichneumonidae*) on an OPM silk nest searching for larvae beneath the nest silk to oviposit. Parasitoid wasps represent natural biological control agents with variable local impact. (B) Mechanical nest removal by vacuuming, illustrating the close proximity of the worker to the nest and the necessity for appropriate personal protective equipment, often requiring the use of aerial work platforms, which entail substantial costs and subsequent thorough decontamination to prevent secondary spread of urticating setae. Photographs were taken during documented field control interventions by trained personnel using standard protective equipment.

Because setae can persist in the environment for years and recurrent seasonal exposure is common, surveillance and targeted control remain important public‐health measures [11, 12, 41, 44, 78, 79, 80, 81]. Intervention is most relevant in high‐exposure settings such as residential areas, schools, playgrounds, and other public spaces, where integrated risk communication and avoidance advice may reduce morbidity. Figure 6 summarises selected examples of mechanical and biological control strategies.

Mechanical nest removal remains the main immediate measure to reduce human exposure, but it requires strict precautions to avoid secondary dispersal of urticating setae [78]. Biological control, particularly early application of *Bacillus thuringiensis* subsp. kurstaki, may reduce larval populations, although effectiveness depends on timing and local ecological conditions [78, 82, 83, 84, 85, 86, 87, 88]. In urban settings, professional monitoring, access restriction to infested areas, and appropriate personal protective equipment for exposed workers are key components of risk reduction [64, 73, 78].

## 4 Discussion: Future Perspectives and Research Directions

Important mechanistic and translational knowledge gaps persist in processionary‐moth–associated disease. Future work should integrate molecular allergology, epithelial and neuro‐immune biology, and exposure science to define clinically relevant endotypes and improve risk stratification. A major limitation of the current literature is the predominance of small, heterogeneous observational studies with limited follow‐up.

Priorities include systematic characterisation of allergenic and pro‐inflammatory setae components, validation of standardised extracts and component‐resolved IgE assays, and harmonisation of basophil activation protocols [10, 46, 47, 48, 62]. Such approaches may help to distinguish genuine IgE‐mediated sensitisation from toxin‐driven irritant mechanisms and to refine diagnostic algorithms.

Longitudinal and multicentre studies are also needed to clarify exposure‐response relationships, sensitisation patterns, and predictors of recurrent or severe disease, including ocular, respiratory, and systemic manifestations [1, 58].

## 5 Conclusion

Processionary‐moth exposure is an increasingly relevant cause of toxic–irritant and allergic disease in Europe. Its clinical spectrum ranges from dermatitis and ocular injury to respiratory symptoms and, rarely, IgE‐mediated systemic reactions including anaphylaxis. Distinguishing toxin‐driven irritant disease from genuine allergic sensitisation is clinically important because latency, phenotype, and risk of systemic involvement differ between endotypes.

Current diagnosis remains primarily clinical and exposure‐based, while standardised extracts and validated molecular diagnostics are still lacking. Table 3 summarises the key clinical take‐home points for allergy specialists, including endotype differentiation, diagnostic orientation, ocular red flags, anaphylaxis risk, and prevention of recurrent exposure. Further research is needed to refine endotype‐specific diagnostics and improve preventive and therapeutic strategies.

**TABLE 3:** Clinical take‐home points for allergy specialists.

**1. Distinct mechanistic endotypes**
Toxic–irritant reactions (mechanical penetration, thaumetopoein‐/TRPV1‐mediated inflammation) vs. IgE‐mediated allergy (sensitisation to Tha p 1/Tha p 2; immediate‐type symptoms; anaphylaxis risk).
**2. Diagnostic orientation through history and latency**
Minutes to 1 h after exposure and cutaneous symptoms resolving within approximately 24 h favour IgE‐mediated reactions; delayed pruritic papules and dermatitis persisting for several days are more consistent with toxic–irritant mechanisms, although clinical overlap may occur.
**3. Phenotype‐guided diagnostic testing**
Skin‐prick testing with non‐standardised in‐house extracts (no validated non‐irritant concentrations; research setting; restricted to experienced centres), component‐resolved IgE (research‐grade), and basophil activation testing may support endotype assignment in selected cases and should be interpreted with caution.
**4. Ocular red flags requiring urgent referral**
Photophobia, visible setae, reduced visual acuity, new floaters, or worsening symptoms despite decontamination warrant same‐day ophthalmologic evaluation to reduce the risk of intraocular migration.
**5. Reported anaphylaxis cases are rare**
Anaphylaxis appears to be rare and is documented mainly in isolated case reports. Patients with previous systemic reactions or unavoidable repeated exposure require counselling, avoidance strategies, and individualised emergency plans (including epinephrine auto‐injectors when appropriate).
**6. Environmental persistence increases recurrence risk**
Setae remain biologically active for years and disperse via wind or contaminated surfaces, making recurrent seasonal symptoms more likely and necessitating anticipatory guidance for patients.

## Author Contributions

Conceptualisation: P.D., T.B. Literature search and data curation: P.D., J.H. Methodology and critical appraisal: T.B., M.P.S., M.S., C.S. Clinical expertise and interpretation: T.B., S.F., M.P.S. Mechanistic and pathological input: C.V., C.S., M.S. Public health and occupational relevance: K.G., W.R. Writing – original draft: P.D., W.R., J.H., T.B. Writing – review and editing: All co‐authors. Visualisation and figures: P.D., J.H., W.R., K.G. Supervision: T.B., M.P.S.

## Funding

This work was supported by a grant from the Federal Ministry of Food and Agriculture (Germany) to TB (FNR #2220NR145X).

## Conflicts of Interest

The authors declare no conflicts of interest.

## Data Availability

Data sharing not applicable to this article as no datasets were generated or analysed during the current study.
